# 5f Covalency Synergistically Boosting Oxygen Evolution of UCoO_4_ Catalyst

**DOI:** 10.1021/jacs.1c10311

**Published:** 2021-12-08

**Authors:** Xiao Lin, Yu-Cheng Huang, Zhiwei Hu, Lili Li, Jing Zhou, Qingyun Zhao, Haoliang Huang, Jian Sun, Chih-Wen Pao, Yu-Chung Chang, Hong-Ji Lin, Chien-Te Chen, Chung-Li Dong, Jian-Qiang Wang, Linjuan Zhang

**Affiliations:** † Key Laboratory of Interfacial Physics and Technology, Shanghai Institute of Applied Physics, Chinese Academy of Sciences, Shanghai 201800, China; ‡ Department of Physics, Tamkang University, Tamsui, New Taipei City 25137, Taiwan, R.O.C.; § Max Planck Institute for Chemical Physics of Solids, Nöthnitzer Strasse 40, Dresden 01187, Germany; ∥ University of Chinese Academy of Sciences, Beijing 100049, China; ⊥ National Synchrotron Radiation Research Center, Hsinchu 30076, Taiwan, R.O.C.

## Abstract

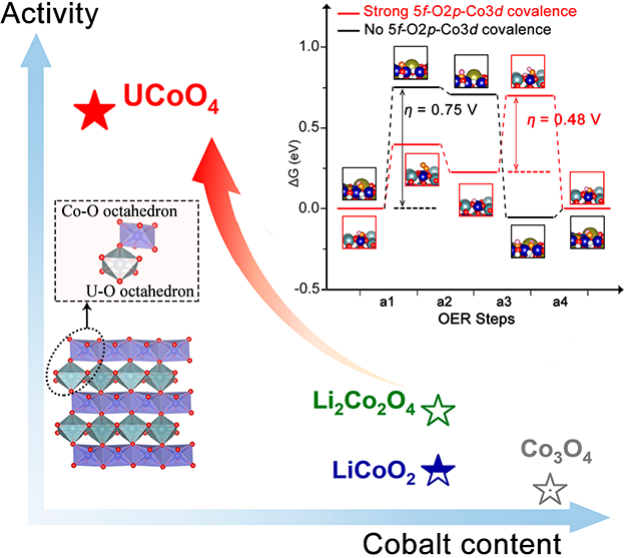

Electronic structure modulation among multiple metal sites is key to the design of efficient catalysts. Most studies have focused on regulating 3d transition-metal active ions through other d-block metals, while few have utilized f-block metals. Herein, we report a new class of catalyst, namely, UCoO_4_ with alternative CoO_6_ and 5f-related UO_6_ octahedra, as a unique example of a 5f-covalent compound that exhibits enhanced electrocatalytic oxygen evolution reaction (OER) activity because of the presence of the U 5f–O 2p–Co 3d network. UCoO_4_ exhibits a low overpotential of 250 mV at 10 mA cm^–2^, surpassing other unitary cobalt-based catalysts ever reported. X-ray absorption spectroscopy revealed that the Co^2+^ ion in pristine UCoO_4_ was converted to high-valence Co^3+/4+^, while U^6+^ remained unchanged during the OER, indicating that only Co was the active site. Density functional theory calculations demonstrated that the OER activity of Co^3+/4+^ was synergistically enhanced by the covalent bonding of U^6+^-5f in the U 5f–O 2p–Co 3d network. This study opens new avenues for the realization of electronic structure manipulation via unique 5f involvement.

## Introduction

Increasing energy demands and environmental issues have prompted intense research on electrochemical water splitting for energy storage and conversion technologies. The sluggish four-electron kinetics of the oxygen evolution reaction (OER) becomes a bottleneck for electrochemical water splitting. Understanding the mechanism and identifying the active sites of the OER are highly important for the design and development of efficient and robust electrocatalysts. Based on the volcano-shaped experimental data, many descriptors affecting the four-electron charge transfer steps on surface-active sites have been proposed, including orbital occupancy close to unity,^1^ O 2p-band center relative to the Fermi level,^2^ charge-transfer energy,^3^ and metal–ligand covalency.^4^ These parameters are usually identified on the basis of the electronic structure of 3d transition metals (TMs) in pristine materials. However, there is increasing experimental evidence that the real active elements undergo electronic structural evolution under OER conditions, including charge, spin, orbital, and structure changes. For example, Co ions undergo a valence state transition from Co^2+^ in the tetrahedral site in Co_3_O_4_ to Co^3+^ of cobalt oxyhydroxide (CoOOH)^5^ and from Co^3+^ to Co^4+^ in spinel Li_2_Co_2_O_4_^6^ and in CoOOH.^7^

In addition to monometallic oxides, some bi- and trimetallic oxide catalysts have been studied, whose OER performance was enhanced by modulating the initial electronic structure of 3d metal active sites. For example, 3d Fe ion introduction can induce a change in the valence state of Co from +2.86 to +3.34 in ZnCo_2_O_4_,^8^ from +2 to +1.96 in CoAl_2_O_4_,^9^ and from +3.5 to +3.4 in perovskite Ba_0.35_Sr_0.65_CoO_3-δ_.^10^ Under applied potentials, the Co valence state changes from ∼1.96 to ∼2.16 in CoFe_0.25_Al_1.75_O_4_ and from ∼2 to ∼2.12 in CoAl_2_O_4_.^9^ The initial valence state of cobalt may affect the evolution of the electronic structure under applied potentials and further affect the charge-transfer behavior. The low valence state of Co ions in the pristine material allows for a high possibility of oxidation during the OER. Despite these reports, the valence state evolution of Co ions during the OER process is less than 1.

Moreover, the modulation of the electronic structure of 3d metal oxide catalysts by using 4d and 5d metal dopants has been widely investigated.^11−14^ Previous studies have revealed that the 4d/5d metal possesses a large d electronic wave function spatial extent, producing versatile electronic structures via the interaction between 3d and 4d/5d orbitals and enhancing the OER activity. However, only a few studies have attempted adjusting the 3d electronic structure via 4f electrons of rare earth metals because of the more localized properties of the 4f orbitals, which do not involve bonding except those of Ce^4+^ ions.^15,16^ Compared to the 4f orbitals, the extended U 5f electrons can directly participate in chemical bonding in actinide compounds.^17^ Recent research shows that uranium and iron oxide heterojunction catalysts exhibit high activity toward water oxidation because of the adjustment of band alignment induced by the “multivalence” of U and Fe ions according to density functional theory (DFT) analysis.^18^ Generally, in uranium oxides, U^6+^-5f and O 2p hybridization is stronger than the hybridization between U^4+^-5f and O 2p,^19^ which implies that U^6+^ modulates the band alignment more easily because of a stronger U^6+^–O covalent bond.^20^ However, it is unclear whether the U 5f–O 2p–Co 3d network can enhance the catalytic activity toward the OER. Based on these considerations, the structurally ordered catalysts formed by combining 3d TM ions and 5f ions are ideal structural models to explore the possible synergistic enhancement of OER activity due to 3d–5f states.

As known previously, edge-sharing CoO_6_ octahedra in catalysts favor OER activity and stability. For example, an increased OER activity is correlated with the surface structural conversion of CoO_6_ from corner-sharing to edge-sharing octahedra in Ba_0.5_Sr_0.5_Co_0.8_Fe_0.2_O_3−δ_ and SrCo_0.8_Fe_0.2_O_3−δ_,^21−23^ while the OER catalysts with the edge-shared and faced-shared networks are stable under OER conditions.^6,24−26^ Herein, we report a new class of metal oxide UCoO_4_ with edge-sharing CoO_6_ and UO_6_ octahedra, as a highly active and durable OER electrocatalyst in an alkaline solution. In UCoO_4_, the initial Co and U ions have Co^2+^ and U^6+^ valence states, respectively, and have fully ordered arrangements of the Co–O–U network. UCoO_4_ shows remarkable OER activity with a low overpotential of 250 mV to reach a current density of 10 mA cm^–2^ and a minimal Tafel slope of 47 mV dec^–1^, which is significantly higher than those of other B-site pure Co oxide catalysts such as LiCoO_2_, Li_2_Co_2_O_4_, Co_3_O_4_, ZnCo_2_O_4_,^27^ and WCoO_4_.^28^ This is unexpected according to descriptors of pristine materials because OER activity is expected at a low Co^2+^ valence state that has a considerable charge transfer energy (Δ) in UCoO_4_.^3^ Operando hard X-ray absorption spectroscopy (XAS) at the Co-K and U-L_3_ edges indicated that the Co ion exhibited an irreversible increase in the valence state at the applied high voltage. Its valence state remains unchanged after switching off voltage, and the U ion has no valence state transition. Soft XAS at the Co-L_2,3_ edge revealed that all Co^2+^ ions in UCoO_4_ are converted to high-valent Co^3+^ and Co^4+^ ions during the OER. U-M_4,5_ indicated strong U 5f and O 2p covalence due to the high valence state of U^6+^ in UCoO_4_. DFT calculations demonstrated that the U 5f–O 2p–Co 3d network facilitated synergistically accelerated OER performance.

## Results and Discussion

**Figure 1:**
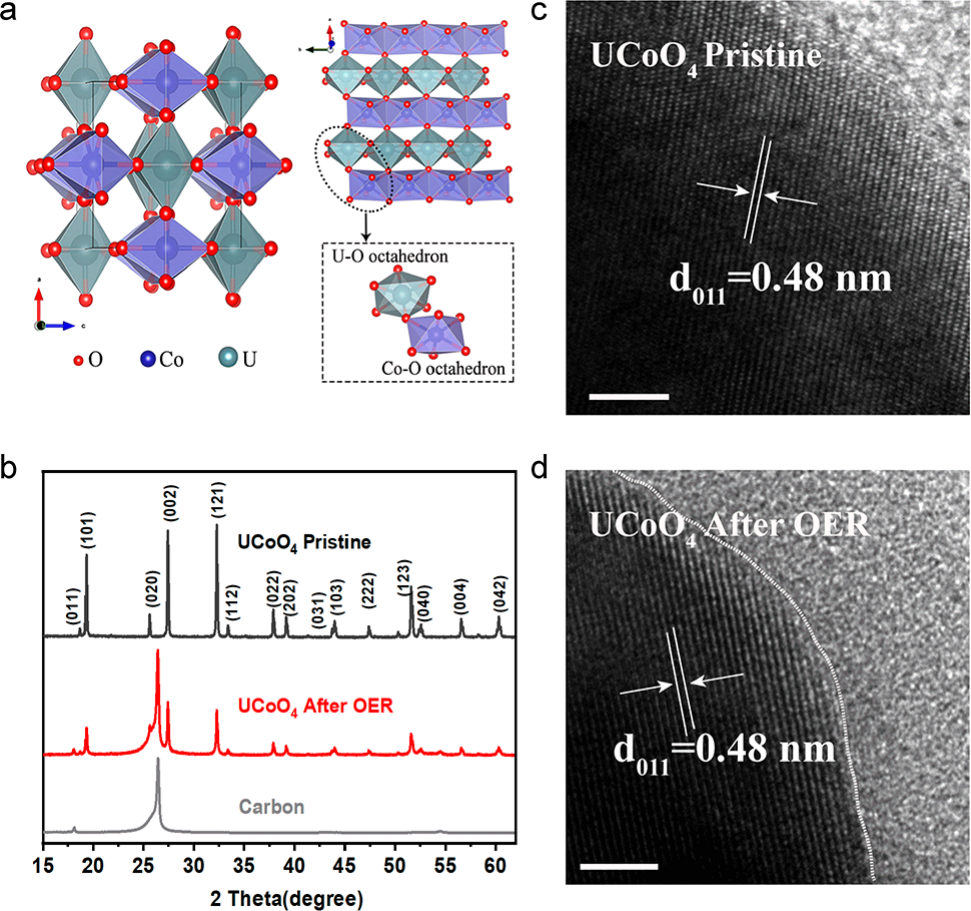
(a) Crystal structure of UCoO_4_. (b) XRD patterns of UCoO_4_ before and after the OER. (c, d) HRTEM images of UCoO_4_ before and after the OER. Scale bar, 5 nm.

Ideal UCoO_4_ compounds contain edge-shared CoO_6_ octahedra separated by order-arranged 5f-related UO_6_ chains (Figure 1a). The crystal structures and morphologies of the synthesized UCoO_4_ samples were investigated through X-ray diffraction (XRD) analysis and transmission electron microscopy (TEM) (Figure 1 and Figure S1a). The diffraction pattern of structurally ordered UCoO_4_ can be indexed to the orthorhombic space group *Imma* with fully ordered Co and U ions in the lattice (Figure 1b and Figure S2); no additional peaks were observed before and after the OER. The formation process of UCoO_4_ was evaluated by conducting differential scanning calorimetry (DSC) and thermogravimetric analysis (TGA) of the precursor and XRD patterns of the sol–gel precursor calcined at different temperatures (Figure S3). We examined the atomic arrangement at the surface regions through high-resolution TEM (HRTEM); the HRTEM images are shown in Figure 1c and d. The lattice fringes with a *d*-spacing of 0.48 nm, corresponding to the (011) planes of UCoO_4_, remain unchanged before and after the OER, demonstrating a stable crystal structure of UCoO_4_. The Co–O–U network collapse was not observed, although the SEM-EDS and ICP-MS results (Table S1) showed small leaching of U during the OER process. Neither surface amorphization in the atomically resolved STEM (Figure S4) nor impurity in the XRD patterns was observed for the UCoO_4_ catalyst before and after the OER. This is because the stability of the crystal structure depends on the metal–O–metal networks, in which the edge-shared networks are widely regarded as a stable configuration.

**Figure 2:**
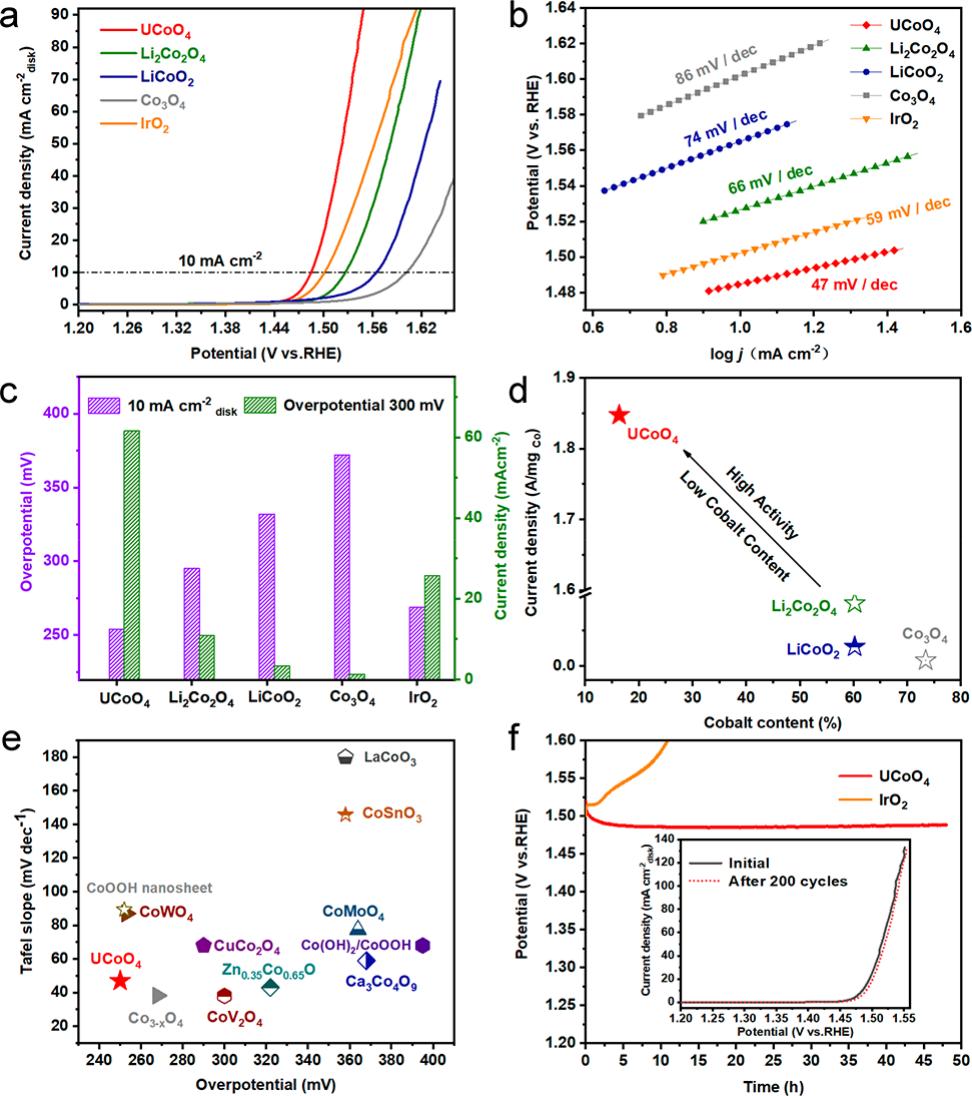
(a) OER LSV curves. (b) Tafel plots. (c) Overpotential required at 10 mA cm^–2^ (left graph in c) and current density at an overpotential of 300 mV (right graph in c). (d) Cobalt mass activities at an overpotential of 300 mV for UCoO_4_, Li_2_Co_2_O_4_, LiCoO_2_, Co_3_O_4_, and IrO_2_. (e) Tafel plots and overpotential at 10 mA cm^–2^ of UCoO_4_ and various Co-based electrocatalysts recently reported. (f) Chronopotentiometric curves at 10 mA cm^–2^ current densities (inset: LSV curves before and after 200 CV cycles).

The electrocatalytic OER activity of the UCoO_4_ nanoparticles in an alkaline electrolyte (1.0 M KOH) was assessed using a typical rotating disk electrode (RDE) technique. All electrode potentials were calibrated with respect to the reversible hydrogen electrode (RHE). Linear sweep voltammetry (LSV) curves were normalized by the geometric area of the electrode, as shown in Figure 2a. The LSV curves show the activity order of UCoO_4_ > IrO_2_ > Li_2_Co_2_O_4_ > LiCoO_2_ > Co_3_O_4_. The OER kinetics were studied using the corresponding Tafel slopes (Figure 2b). The lowest Tafel slope (47 mV dec^–1^) was obtained for UCoO_4_, while the other catalysts showed significantly higher values, verifying the fast intrinsic OER catalytic kinetics of UCoO_4_. The UCoO_4_ catalyst exhibited a low overpotential of 250 mV to reach a current density of 10 mA cm^–2^, outperforming the benchmark IrO_2_ catalyst (269 mV) and other Co-related catalysts (332 mV for LiCoO_2_, 295 mV for Li_2_Co_2_O_4_, and 372 mV for Co_3_O_4_). At an overpotential of 300 mV (+1.53 V vs RHE), the specific OER activity of UCoO_4_ was approximately 2.4 times higher than that of IrO_2_ nanoparticles and nearly 51.3 times higher than that of typical Co_3_O_4_ OER catalysts and greatly exceeded that of LiCoO_2_ and Li_2_Co_2_O_4_ nanoparticles (Figure 2c). Figure 2d shows the cobalt mass activity at an overpotential of 300 mV for the UCoO_4_, Li_2_Co_2_O_4_, LiCoO_2_, and Co_3_O_4_ catalysts. UCoO_4_ exhibited the highest current density of 1.85 A mg^–1^, approximately 20, 67, and 231 times higher than those of the Li_2_Co_2_O_4_, LiCoO_2_, and Co_3_O_4_ nanoparticles, respectively. The electrochemical active surface area (ECSA) results were obtained to evaluate the activity of UCoO_4_ (862 cm^2^) and Li_2_Co_2_O_4_ (598 cm^2^), demonstrating that UCoO_4_ exhibits more catalytically active sites than the reference Li_2_Co_2_O_4_ catalyst (Figure S5). To compare the intrinsic OER activity, the geometric current density was normalized by ECSA. As shown in Figure S6, UCoO_4_ has higher intrinsic activity than the reference Li_2_Co_2_O_4_ catalyst. The overpotential and Tafel slope of UCoO_4_ represent one of the best levels achieved for unitary Co-based OER electrocatalysts in alkaline solutions (Figure 2e and Table S4).^29−35^ Furthermore, stability is one of the most important parameters of electrocatalysts. The chronoamperometry curve showed almost no change during the 48 h electrocatalytic process at 10 mA cm^–2^. After 200 CV cycles between 1.20 and 1.50 V (vs RHE) at a scan rate of 10 mV s^–1^, the overpotential of UCoO_4_ at a current density of 10 mA cm^–2^ only increases by 2 mV (the inset of Figure 2f), confirming the long-term stability of UCoO_4_.

**Figure 3:**
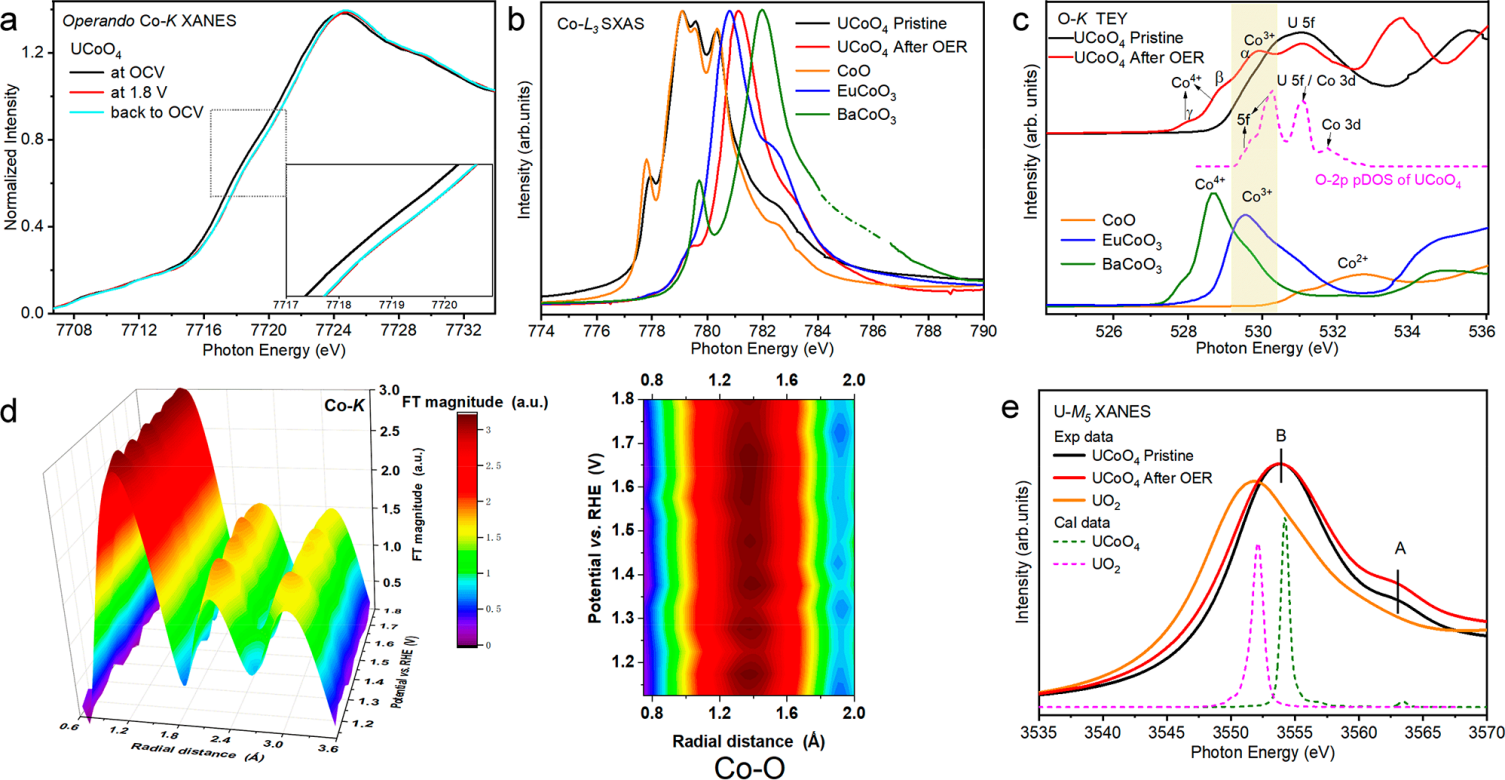
(a) Co-K edge operando XANES spectra for UCoO_4_ at OCV, 1.8 V, and after the OER back to OCV. Inset: Enlarged view of the dotted box. The applied voltage is referenced to RHE. (b) Co-L_3_ ex situ SXAS spectra of UCoO_4_ before and after the OER, together with the spectra of CoO, EuCoO_3_,^36^ and BaCoO_3_^37^ with Co^2+^, Co^3+^, and Co^4+^ as references, respectively. (c) O-K SXAS spectra of pristine UCoO_4_ and after the OER collected in the TEY mode. The magenta dotted line corresponds to the calculated O 2p projected density of states (pDOS) of UCoO_4_. The experimental spectra of CoO, EuCoO_3_, and BaCoO_3_ are presented as references. (d) 3D operando Fourier transforms of *k*_3_-weighted EXAFS spectra at the Co-K edge as a function of applied potential, along with corresponding enlarged 2D contour plots of Co–O. The EXAFS data are not corrected for phase shift. (e) U-M_5_ XANES experimental spectra of UCoO_4_ before and after the OER and UO_2_ as the reference, together with calculated spectra of U^4+^ (dashed magenta) and U^6+^ (dashed olive).

The real active sites and valence states of the active metal ions in UCoO_4_ were examined through fast operando XANES at the Co-K and U-L_3_ edges during the OER. Figure 3a shows the Co-K XANES spectra of UCoO_4_ as a function of the applied voltage. Compared to the initial state conditions at open circuit voltage (OCV), the Co-K absorption edge shifted to a higher energy level under OER conditions at 1.8 V. This indicates an increase in the Co oxidation state and demonstrates that the OER-active Co ions in UCoO_4_ serve as active sites. However, the valence state of U remains unchanged in the UCoO_4_ catalyst before and after the OER according to our XAS measurements at all U-L_3_ (Figure S7), M_5_ (Figure 3e), and N_5_ (Figure S8) edges. In addition, pure U oxides displayed negligible OER activity (Figure S9). These data support that U is OER inactive. The energy shift at the Co-K edge was only 0.35 eV. Using the relationship between the energy shift and Co oxidation state^38−42^ and considering the presence of Co^2+^ in pristine UCoO_4_ (Figure 3b, discussed below), we obtained an estimated Co valence state of +2.15 for UCoO_4_ after the OER, which could not explain the high OER activity observed for this catalyst. The reason is that the reaction layer depth strongly reduces with decreasing Co valence state.^4^ For UCoO_4_, we expect to see only <30% signal from the reacted layers because of the large particle size (∼39 nm, Figure S10) against the ≤2 nm reaction layer (Figure S11).^4^ When the applied potential was removed, the energy position of the Co-K edge remained unchanged, retaining that under the OER conditions, which is consistent with the CV results (Figure S12). This is a nice case known for Li_2_Co_2_O_4_^6^ because the same information about the electronic structure under the OER can be obtained after the OER by using ex situ surface-sensitive soft XAS (SXAS) spectra in the TEY mode at the Co-L_2,3_ and O-K edges (2–4 nm exploring depth), which can provide detailed information about the valence states,^37,43^ spin states,^36,44^ local environments^45^ of the Co ions, and the unoccupied O 2p states.^34^

The electronic structure of UCoO_4_ was further examined using ex situ Co-L_3_, O-K, and U-M_5_ XAS. The multiplet spectral feature and energy position of the Co-L_3_ SXAS spectrum of pristine UCoO_4_ in Figure 3b are very similar to those of CoO, indicating a Co^2+^ state with local octahedral coordination. After the OER, the Co-L_3_ SXAS spectra shifted to a higher energy than that of EuCoO_3_, suggesting mixed Co^3+^ and Co^4+^ in UCoO_4_ after the OER, such as Na_*x*_CoO_2_.^46^ Co^3.2+^ was observed in UCoO_4_ after the OER (Figure S13), which was due to U leaching (Table S1). This phenomenon has been intensively studied.^10^Figure 3c shows the O-K SXAS spectra of UCoO_4_ before and after the OER in the TEY mode. The pre-edge peaks below 533 eV in the Co oxides are due to the unoccupied O 2p orbitals hybridized with the Co 3d states. A lower energy shift and an increase in the spectral weight of the pre-edge peaks with increasing valence state from Co^2+^ in CoO to Co^3+^ in EuCoO_3_ and further to Co^4+^ in BaCoO_3_ were observed. In pristine UCoO_4_, the peak at 531 eV is mainly attributed to the U 5f states mixed with the O 2p states because Co^2+^ has a very weak contribution from CoO (Figure 3c), as demonstrated by our DFT calculations (magenta). After the OER, three new spectral features, α, β, and γ, appear similar to those of Na_0.75_CoO_2_^46^ and Li_2_Co_2_O_4_,^6^ wherein peak α is assigned to the unoccupied e_g_ orbitals of Co^3+^, while peaks β and γ are attributed to the unoccupied t_2g_ and e_g_ orbitals of Co^4+^, respectively. Both Co-L_3_ and O-K SXAS spectra demonstrate a significant increase in the valence of Co from +2 to +3.2 after the OER over UCoO_4_. Additionally, we also carried out the XPS analysis for UCoO_4_ before and after OER (Figure S14), which supports the Co^2+^ ions in pristine UCoO_4_ were converted to high-valence Co^3+/4+^,^47^ and U^6+^ remained unchanged during the OER. The local coordination environments of the OER-active Co ions were also investigated by the operando extended XAFS experiments. The 3D Fourier transform patterns of the Co-K edge spectra as a function of applied potential are shown in Figure 3d. However, only a small decrease in the Co–O bond length could be observed with increasing applied voltages (Figure S15 and Table S2), reflecting the small reaction depth against the considerable exploring depth of the hard X-ray.

Next, we verified the presence of U^6+^ in pristine UCoO_4_ before and after the OER to meet the charge-balance requirement. The U-M_5_ XANES spectrum of UCoO_4_ shifted to a higher energy of ∼2 eV compared to that of UO_2_ (collected in the fluorescence mode in Figure 3e and the transmission mode in Figure S16), in agreement with the U-N_5_ edge of UCoO_4_ (Figure S8), thereby confirming the presence of U^6+^. In addition, a weak high-energy shoulder above the main peak was observed in the UCoO_4_ spectrum (marked by A) in Figure 3e, which is characteristic of the U 5f–O 2p covalence. A similar feature was observed at the M_4,5_ edge of tetravalent rare earth metals because of the 4f-ligand 2p covalence.^48−50^ Furthermore, we simulated the experimental U-M_5_ spectrum (Figure 3e) using the Anderson impurity model for the U^6+^ and U^4+^ systems, with the same parameters as refs (19) and (51). The main and satellite peaks at the U-M_5_ edge of UCoO_4_ (dashed green) can be attributed to the 3d^9^5f^2^L and 3d^9^5f^1^ final states, respectively. No satellite peak for U^4+^ indicates weak 5f–O 2p covalence because the charge transfer energy (Δ) decreases from 6.5 eV for U^4+^ to 3.5 eV for U^5+^ and further to 0.5 eV for U^6+^.^19^ The strong U 5f–O 2p–Co 3d covalence was also confirmed by the calculated O 2p pDOS pattern in UCoO_4_ (dashed magenta in Figure 3c). After the OER (red line), the increased character of the O 2p states near the Fermi level enables the injection/extraction of electrons from oxygen sites, thus favoring OER activity.^1^ This finding confirms the synergistic effect of the 5f states in valence bonding although the U ion is OER inactive.

**Figure 4:**
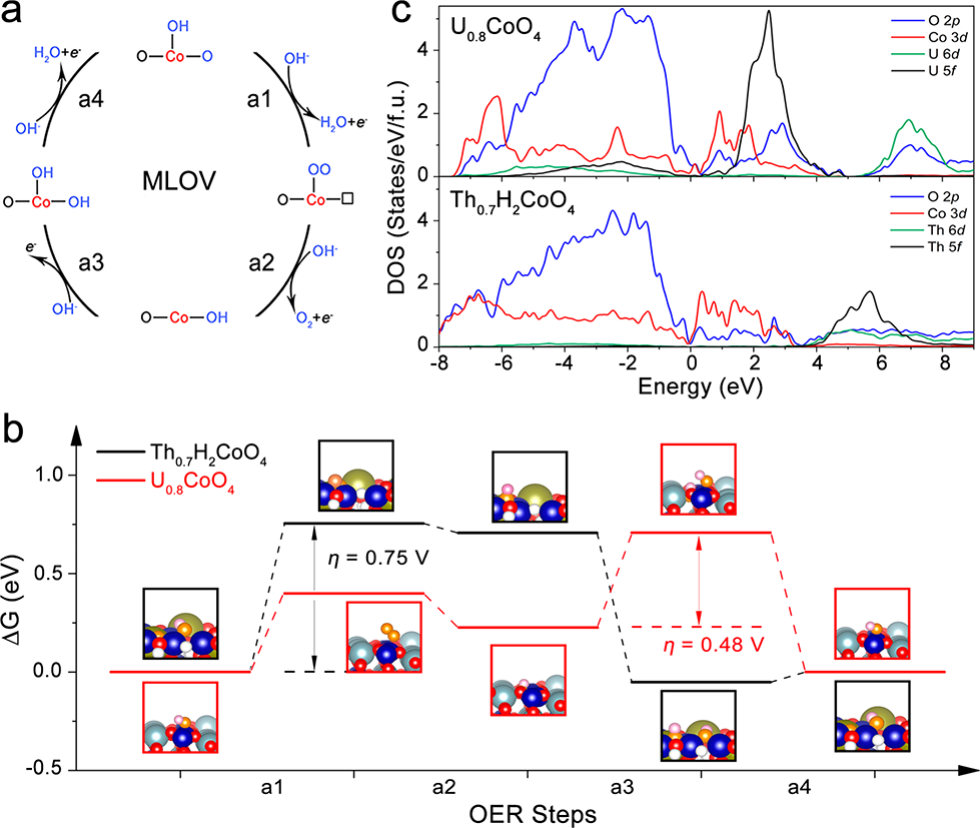
Schematic OER mechanisms involving four concerted proton–electron transfer steps. (a) MLOV scenario. (b) Free energies at *U*_RHE_ = 1.23 V of OER steps for Th^4+^_0.7_H_2_Co^3.2+^O_4_ and U^6+^_0.8_Co^3.2+^O_4_ structural models. (c) Projected density of states of U_0.8_CoO_4_ and Th_0.7_H_2_CoO_4_. The Fermi levels are set to 0 eV.

We investigated the synergistic effects of the 5f and 3d orbitals in the UCoO_4_ system using the first-principles DFT calculations. The (001) plane was used to model the surface reaction pathways (Figure S17). We first calculated the charge-density differences and Bader charges of UCoO_4_ and U_0.8_CoO_4_, as shown in Figure S18. For the Co atoms in U_0.8_CoO_4_, the charge density is reduced, and the Bader charge is increased, indicating that the valence state of Co is significantly increased to Co^3.2+^ states. To demonstrate the argument on the active sites of Co and U atoms, we calculated the electrochemical OER on the surface U (Figure S19) and Co atoms (Figure 4) in U_0.8_CoO_4_. For U active sites, we considered the metal-site adsorbate evolution mechanism (MAE), which is the conventional approach. The rate-limiting step for U is the adsorption of *O, and the overpotential is 0.70 V in U_0.8_CoO_4_. This large overpotential of U atoms demonstrates that the U ion is OER inactive. For Co oxides with high-valent Co^3+/4+^ ions, the metal-and-lattice-oxygen-vacancy-site (MLOV) mechanism has been proven to be the most favorable scenario,^6^ wherein the adsorbates are located at both TM sites and lattice oxygen vacancy sites (Figure 4a). Based on the MLOV mechanism, the Gibbs free energy difference (Δ*G*) of the electrochemical OER in U_0.8_CoO_4_ is shown in Figure 4b. The rate-limiting step for Co is the adsorption of *OH, and the overpotential is 0.48 V in U_0.8_CoO_4_, which is lower than that of single Co as an active site known previously.^6,12,52^ This lower overpotential is due to the participation of U-5f, which can be verified on the basis of the charge-density differences of four adsorbed species in Figure S20. The accelerated electron transfer involves the neighboring U atom, which activates the lattice oxygen and makes the OER thermodynamically favorable. In addition, the pDOS of U_0.8_CoO_4_ shows an obvious overlap among U 5f, O 2p, and Co 3d near *E*_f_ (Figure 4c), which promotes the OER activity. To confirm such a low overpotential uniquely from the 5f participating bonding from the U^6+^ state, we also calculated a model system, Th^4+^_0.7_H_2_CoO_4_, with the same 5f^0^ configuration and crystal structure but with negligible 5f–O 2p covalence.^53^ In Figure 4c, the Th 5f states are located at much higher energies and have no overlap with O 2p and Co 3d close to *E*_f_ as Co ions have the same valence (+3.2 in both Th_0.7_H_2_CoO_4_ and U_0.8_CoO_4_), the only difference between these two systems is whether the 5f orbital participates in bonding with the Co 3d/O 2p orbitals. Furthermore, the participation of the 5f orbital in covalent bonding leads to an increase in the ratio of O 2p pDOS to Co 3d pDOS near *E*_f_, from ∼1.4 in Th_0.7_H_2_CoO_4_ to approximately 3.0 in U_0.8_CoO_4_. All features in O 2p pDOS indicate that the oxidation of lattice oxygen becomes thermodynamically favorable if strong U 5f is strongly mixed with Co 3d/O 2p, as confirmed from the Δ*G* analysis. Moreover, in Figure 4b, the overpotential for Th_0.7_H_2_CoO_4_ is 0.75 V, higher than 0.48 V for U_0.8_CoO_4_, confirming the important role of 5f participation toward OER activity enhancement. The adsorption of *OH at lattice oxygen vacancy sites in U_0.8_CoO_4_ is no longer a rate-limiting step, unlike that in Th_0.7_H_2_CoO_4_, because the lattice oxygen is further activated by 5f participating in covalent bonding. The higher O 2p pDOS near *E*_f_ in U_0.8_CoO_4_ than that in Th_0.7_H_2_CoO_4_ in Figure 4c is considered to be the contributor to OER activity enhancement.^39^ Thus, our theoretical results prove that OER activity can be synergistically enhanced through strong U 5f–O 2p covalent mixing.

## Conclusions

Herein, we demonstrated that UCoO_4_, featuring unique edge-sharing CoO_6_ and UO_6_ octahedra, exhibited extraordinary electrocatalytic activity toward the OER. Electrochemical measurements showed that the UCoO_4_ catalyst displayed a low overpotential of ∼250 mV at a current density of 10 mA cm^–2^ in 1 M KOH solution, demonstrating the highest OER activity among unary cobalt oxides ever reported. In the UCoO_4_ catalyst, although U is an OER-inactive ion, strong U 5f–O 2p covalence can lead to synergistically enhanced OER activity because of the Co–O–U network originating from an extremely high oxidation state U^6+^. This is a particular case for U^6+^ systems because the 5f orbitals in U^4+^ and U^5+^ compounds have insignificant covalent mixing with the O 2p states. Thus, our proof-of-concept study not only promotes the development of a highly active and stable OER electrocatalyst but also opens up new approaches to realize electronic structure modulation via unique 5f electron involvement, enabling the transformation of nuclear power plant waste into valuable products.

## Experimental Section

### Synthesis of Catalysts

UCoO_4_ nanoparticles were synthesized via a facile sol–gel method. An aqueous solution of 1 mmol of cobalt nitrate and 1 mmol of uranyl nitrate was dissolved in 40 mL of water, followed by the addition of 2 mmol of citric acid and 2 mmol of urea. After decomposition at 180 °C for over 12 h, the obtained gel was sintered in a tube furnace under high-purity air, and the sintering temperature was (1) increased from room temperature to 400 °C at 1 °C/min and held for 2 h; (2) increased further from 400 °C to 700 °C at 2 °C/min and held for 24 h; and (3) allowed to cool naturally to room temperature.

### Material Characterization

The crystal structures of the prepared catalysts were characterized by powder XRD on a Bruker D8 Advance X-ray diffractometer using a Ni-filtered Cu Kα radiation source at 40 kV and 40 mA. TEM images were acquired using an FEI Tecnai G2 F20 electron microscope operating at 200 kV. Before being transferred to the TEM chamber, the samples dispersed in ethanol were deposited onto a carbon-coated copper grid and quickly moved into a vacuum evaporator. Co-K edge operando XAS was performed at beamline 44A of the National Synchrotron Radiation Research Center (NSRRC). The electron storage ring was operated at 3.0 GeV with a constant current of ∼500 mA. The corresponding data were collected in transmission mode. To have a quality spectrum, we applied the Quick-XAS mode with 120 spectra within 1 min. To get the high quality of EXAFS spectrum, we sum up 120 spectra within 1 min if they are not distorted by bubbles. U L_3_-edge operando XAS was performed at BL14W1 of the Shanghai Synchrotron Radiation Facility (SSRF). The electron storage ring was operated at 3.5 GeV with a maximum current of ∼210 mA. Ex situ XAS experiments at the Co-L_3_ edge, O-K, and U-N_5_ edges were carried out at the 11A beamline of the NSRRC. U M_5_-edge XAS spectra were collected in the fluorescence mode at 16A1 of NSRRC and the transmission mode using an in-house laboratory-based X-ray absorption spectrometer with a Si (220) spherical bent crystal analyzer.

### Electrochemical Measurements

Electrochemical measurements were performed using a three-electrode system connected to an Autolab PGSTAT302N electrochemical workstation at room temperature. A rotating disk working electrode (WE) made of glassy carbon (GC, PINE, 5 mm diameter, 0.196 cm^2^) was used. Catalyst powder (5 mg) and carbon powder (Vulcan XC72, 5 mg) were dispersed in a mixture of water (750 μL), ethanol (250 μL), and Nafion solution (40 μL) under continuous ultrasonication. Subsequently, 8 μL of the catalyst ink was transferred to a polished GC electrode and allowed to dry naturally. A Hg/HgO electrode in 1 M KOH aqueous solution was used as the reference electrode, and a platinum wire counter electrode was placed in a fritted glass tube. The electrolyte was an aqueous solution of 1 M KOH, saturated with oxygen, and bubbled for 30 min before each test. OER polarization curves were obtained at a scan rate of 5 mV s^–1^ in O_2_-saturated 1 M KOH solutions. All potential versus RHE is in line with the equation *E*(RHE) = *E*(Hg/HgO) + 0.059pH + 0.096 V. The pH of the 1 M KOH aqueous solution at room temperature was 13.60.

### Density Functional Simulations

The present calculations employ the Vienna Ab initio Simulation Package (VASP)^54,55^ implementation of DFT in conjunction with the projector augmented wave (PAW) formalism. The exchange–correlation term was modeled using the general gradient approximation (GGA) with Perdew–Burke–Ernzerhof (PBE).^56^ For the U_0.8_CoO_4_ and Th_0.7_H_2_CoO_4_ systems, all DFT calculations were performed with VASP in conjunction with the PAW formalism, and the exchange–correlation term was modeled using GGA with PBE. The H 1s^1^, O 2s^2^2p^4^, Co 4s^2^3d^7^, U 6s^2^6p^6^7s^2^6d^1^5f^5^, and Th 6s^2^6p^6^7s^2^6d^2^ states were treated as valence electrons. The electronic wave functions are expanded in plane waves using an energy cutoff of 400 eV. The force and energy convergence criteria were set to 0.02 eV/Å and 10^–5^ eV, respectively, and the Monkhorst–Pack *k*-point meshes are 2 × 2 × 1. The value of *U*_eff_ (=*U* – *J*) of the Co 3d*,* U 5f, and Th 5f states was set to 3.52, 4.00, and 4.00 eV, according to previous work.^52,57,58^ For U_0.8_CoO_4_ and Th_0.7_H_2_CoO_4_, we used the 1 × 1 primitive cell (lattice constants are *a* = *c* = 6.497 Å, *b* = 6.952 Å) to build periodic slab models with 12 Co sites per surface, with two layers at the bottom that were fixed during relaxation. For Th_0.7_H_2_CoO_4_, we adopted the optimized 1 × 1 primitive cell by adding two H atoms in the interstitial space and then built periodic slab models with eight Co sites per surface, with two layers at the bottom that were fixed during the relaxation. The thickness of the vacuum spacing was ∼15 Å in the *z*-direction.

### Free Energy Calculations

The elementary steps of MLOV mechanisms are listed below:
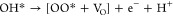
1
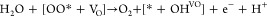
2
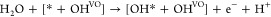
3
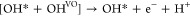
4The Gibbs free energy changes (Δ*G*) were calculated by the following equations:
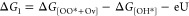
5
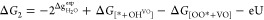
6
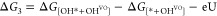
7
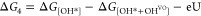
8where *U* is the potential measured against RHE at standard condition (*T* = 298.15 K, *P* = 1 bar, pH = 0) and  is the experimental Gibbs free energy of formation of water molecules. The Δ*G* of these intermediates includes zero-point energy (ZPE) and entropy corrections (listed in Table S3) according to Δ*G*_i_ = Δ*E*_i_ + ΔZPE_i_ – *T*Δ*S*_i_, where the energy differences Δ*E*_i_ are calculated with respect to H_2_O and H_2_ (at *U* = 0 and pH = 0). The theoretical overpotential is defined as the lowest potential at which all reaction steps are thermodynamically downhill.

## Supporting Information Available

Detailed methods, experiment process, and additional XRD, TEM, XAS, cyclic voltammetry, LSV, and DFT data (PDF)
