# Supplementary material for: 5f Covalency Synergistically Boosting Oxygen Evolution of UCoO4 Catalyst
Source: J Am Chem Soc. 2021 Dec 8;144(1):416–23. doi: 10.1021/jacs.1c10311 (PMC8759065; doi:10.1021/jacs.1c10311)
Supplement: Supplementary file 1 — ja1c10311_si_001.pdf [file ja1c10311_si_001.pdf]

5f Covalency Synergistically Boosting Oxygen Evolution of $UCoO_4$ Catalyst

Xiao Lin[a], Yu-Cheng Huang[b], Zhiwei Hu[c*], Lili Li[a], Jing Zhou[a], Qingyun Zhao[a], Haoliang Huang[a], Jian Sun[a,d], Chih-Wen Pao[e], Yu-Chung Chang[e], Hong-Ji Lin[e], Chien-Te Chen[e], Chung-Li Dong[b], Jian-Qiang Wang[a,d*] and Linjuan Zhang[a,d*]

[a] Dr. X. Lin, Dr. L. Li, Dr. J. Zhou, Q. Zhao, Dr. H. Huang, J. Sun, Prof. J.-Q. Wang, and Prof. L. Zhang

Key Laboratory of Interfacial Physics and Technology, Shanghai Institute of Applied Physics, Chinese Academy of Sciences, Shanghai 201800, China

Email: wangjianqiang@sinap.ac.cn; zhanglinjuan@sinap.ac.cn.

[b] Y-C. Huang, and Prof. C. Dong

Department of Physics, Tamkang University, Tamsui, New Taipei City 25137, Taiwan, R.O.C

[c] Dr. Z. Hu

Max Planck Institute for Chemical Physics of Solids, Nöthnitzer Strasse 40, Dresden 01187, Germany

Email: zhiwei.hu@cpfs.mpg.de

[d] J. Sun, Prof. J.-Q. Wang, and Prof. L. Zhang

University of Chinese Academy of Sciences, Beijing 100049, China

[e] Dr. C.-W. Pao, Dr. Y.-C. Chang, Prof. H.-J. Lin, Prof. C.-T. Chen

National Synchrotron Radiation Research Center, Hsinchu 30076, Taiwan, R.O.C

Experimental section

Synthesis of reference samples. $Li_2Co_2O_4$ was also synthesized using sol-gel method. An aqueous solution of 1 mmol cobalt nitrate and 1mmol lithium nitrate was dissolved in 40 mL water, followed by the addition of citric acid, and urea. The solution was vigorously stirred for 2 h and allowed to stand overnight in an open Petri dish at 80 °C, a gel was obtained. The as-prepared gel was first decomposed at 180 °C for over 12 h, then annealed at 350 °C for 48 h under high purity air. $LiCoO_2$ was synthesized using the same method, but the annealed temperature is 700 °C and stayed for 24 h under high purity air. Co_3O_4 were synthesized also using the similar process as that of $UCoO_4$, but without the addition of uranyl nitrate. In addition, the IrO_2 (99.99% metals basis) powder used in this study was purchased from Alfa Aesar.

Formation process of the synthesized $UCoO_4$. To understand the formation process of $UCoO_4$, we also carried differential scanning calorimetry (DSC) and thermogravimetric (TGA) analyses of precursor as shown in Figure S3a using a DSC-

TGA Discovery SDT 650 with a heating rate of 10°C/min in a continuous air flow (100 ml/min). It was found that the DSC cure exhibited an endothermic peak at 84℃ and 280℃, which was due to the dehydration and decomposition of cobalt nitrate and uranyl nitrate [*J. Alloys Compd.* 2009, 468, 443], respectively. At 366℃ a broad peak is observed which is due to the combustion of the uncomplexed citric acid and urea [*Chem. Mater.* 2000, 12, 2763]. The uranium cobalt oxide phase formation is observed clearly around 608℃, which indicates that the sample undergo a very rapid transformation without the need of atomic diffusion. To further to explore possible intermediate stages of the formation of $UCoO_4$, we also studied the XRD patterns of sol-gel precursor calcined at different temperatures as shown in Figure S3b. At 500℃, the CoU_3O_{10} phase is observed and additional Co mainly exists in the form of Co_3O_4 with low-crystalline. At 600℃, the CoU_3O_{10} exhibits much narrower diffraction lines. Subsequent heating to higher temperatures at 700℃, well-crystalline $UCoO_4$ phase was obtained. Based on above results, there is no conversion of uranyl compounds to UO_3 at 600 °C in this sol-gel synthesis process. Instead, it generates the CoU_3O_{10} phase at 500 °C and then converts to $UCoO_4$ phase at 700 °C.

Electrochemical surface area measurements of the synthesized $UCoO_4$ and $Li_2Co_2O_4$. The electrochemical surface area (ECSA) results were obtained as following. Based on the CV curves of each sample with different scan rates (20, 40, 60, 80 and 100 mV s^{-1}) as shown in Figure S5, the corresponding C_{dl} of $UCoO_4$ and reference sample $Li_2Co_2O_4$ were calculated by linear fitting to be 34.50 and 23.91 mF, respectively. The ECSA of the sample is calculated according to the equation ECSA = C_{dl} / C_s, where ECSA is proportional to C_{dl} and C_s is a constant whose value is reported to be 0.040 mF cm^{-2}. It was found that the ECSA values of $UCoO_4$ (862 cm^2) was higher than that of the $Li_2Co_2O_4$ (598 cm^2). This result demonstrates that $UCoO_4$ could expose more catalytical active sites than the reference sample $Li_2Co_2O_4$, thus exhibit more excellent catalytic activity for OER.

Structural characterization. The Evolution of the U/Co ratio in $UCoO_4$ sample before and after OER reaction at 10 mA cm^{-2} current densities determined by the inductively coupled plasma mass spectrometry (ICP-MS, PerkinElmer NexION 300X) and Scanning electron microscopy and energy dispersive X-ray spectroscopy (SEM-EDS) analyses were performed on a Zeiss CB 540. X-ray photoelectron spectrometry equipped with an ESCA-LAB 250Xi analyzer: first energy scale calibration, adjusting C 1s peak to 284.6 eV. High-angle annular dark-field scanning transmission electron microscopy (HAADF-STEM) characterizations were conducted in a 300kV double aberration-corrected Titan Themis Z electron microscope. The convergent angle of the electron beam for HAADF-STEM and EDS was 25 mrad.

Results section

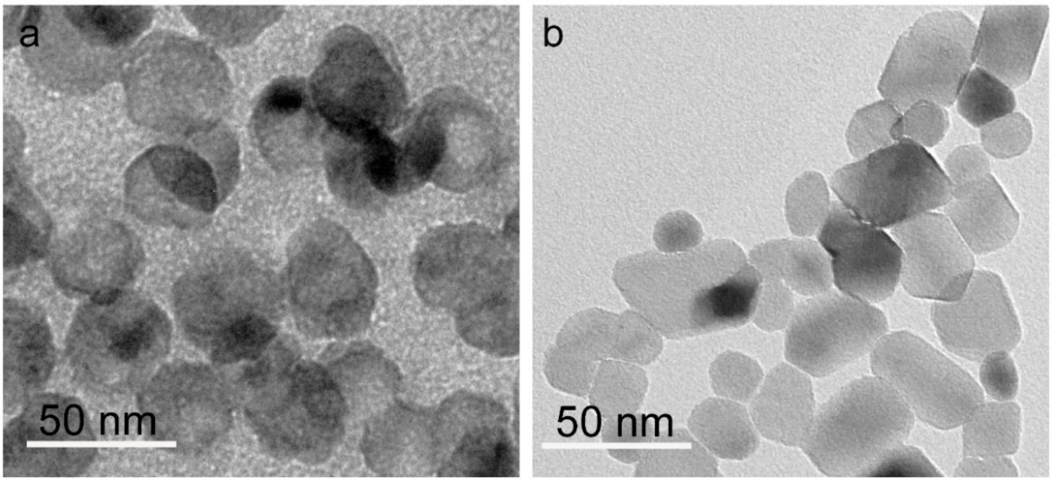

Figure S1. TEM images of **(a)** $UCoO_4$ and **(b)** $Li_2Co_2O_4$.

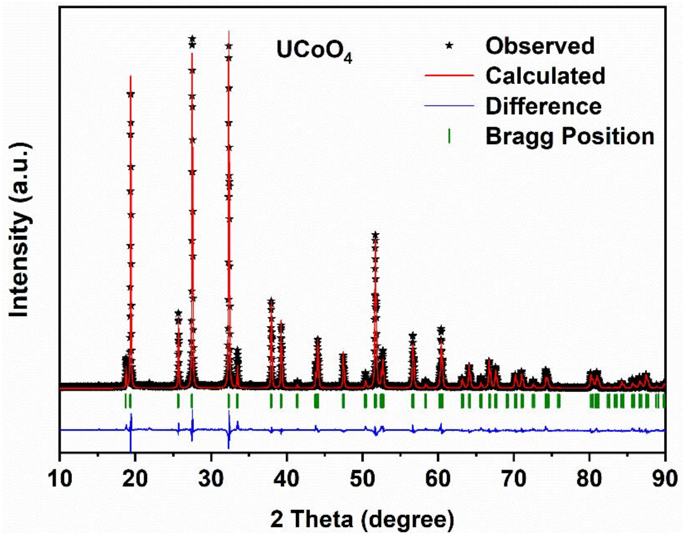

Figure S2. Rietveld refined $UCoO_4$ powder XRD pattern. The experimental data and calculated patterns correspond to black stars and red line, respectively, and allowed Bragg reflections in olive vertical bars, and the difference curves in blue.

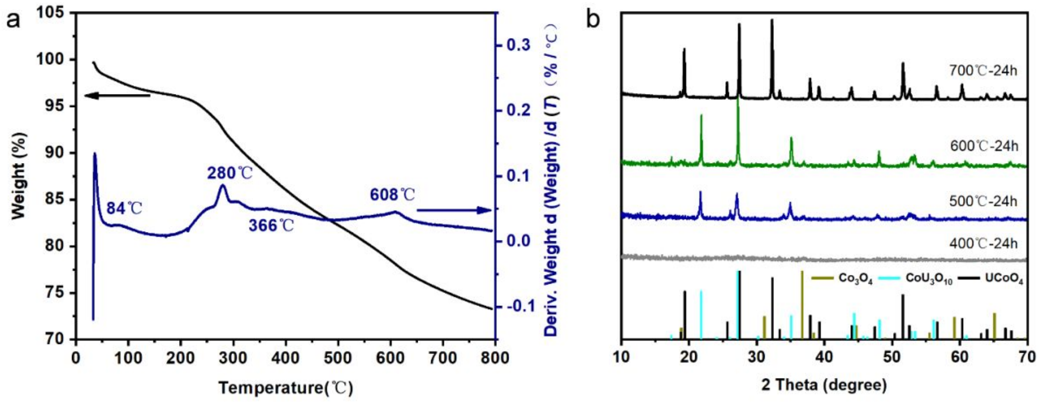

Figure S3. **(a)** DSC-TGA of the preparation of $UCoO_4$ precursor carried out in dynamic air and **(b)** XRD patterns of $UCoO_4$ precursor calcined at different temperatures for 24 hours.

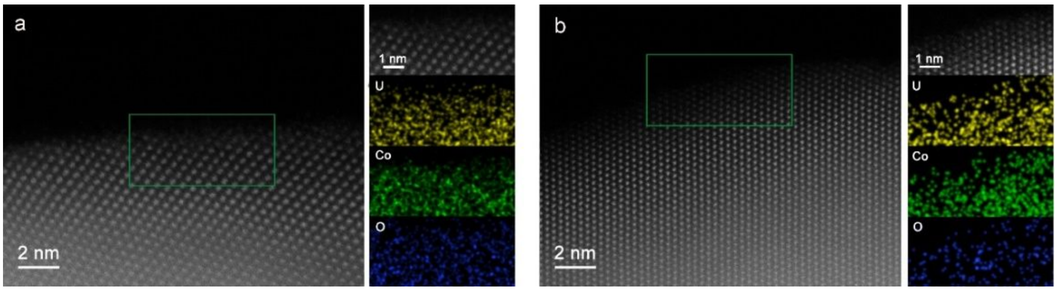

Figure S4. Atomically resolved STEM images and U, Co, and O elemental mapping distribution of $UCoO_4$ pristine **(a)** and after OER **(b)**.

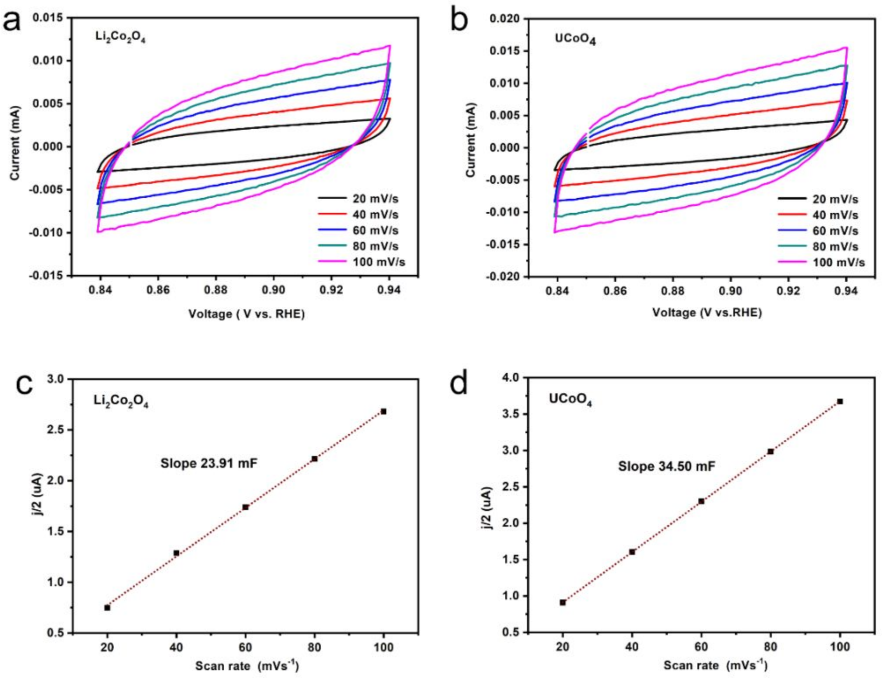

Figure S5. (a, b) Cyclic voltammograms (CVs) for $UCoO_4$ and reference sample $Li_2Co_2O_4$ at the scan rates of 20, 40, 60, 80 and 100 mV s^{-1}, respectively. **(c, d)** Current densities determined at a potential of 0.89 V (V vs. RHE) as a function of scan rate for UCoO4 and reference sample $Li_2Co_2O_4$, respectively.

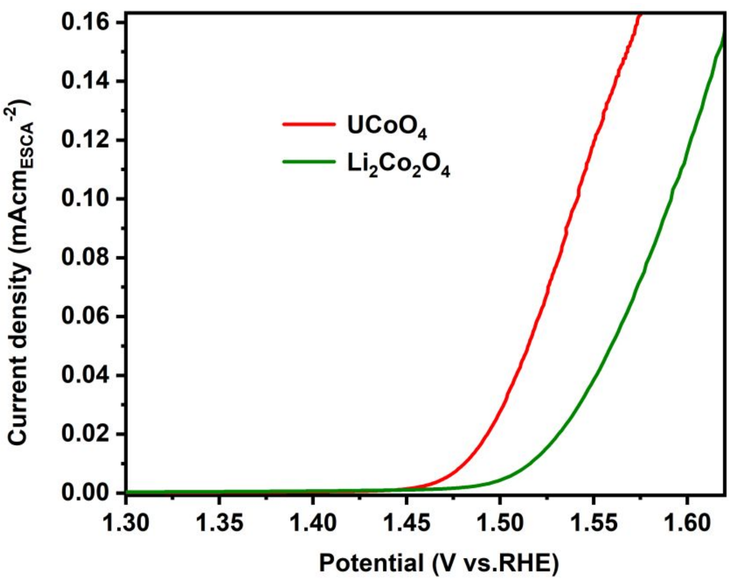

Figure S6. Geometric current density normalized by ECSA of $UCoO_4$ and reference $Li_2Co_2O_4$.

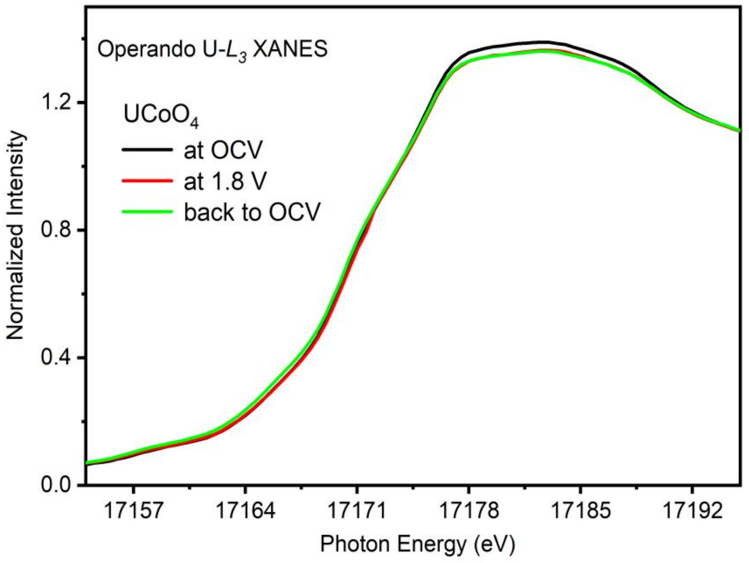

Figure S7. U-L_3 edge XANES spectra for $UCoO_4$ at OCV (black line) and 1.8 V (red line) and after reverting the OCV (cyan line). The applied voltage is referenced to RHE.

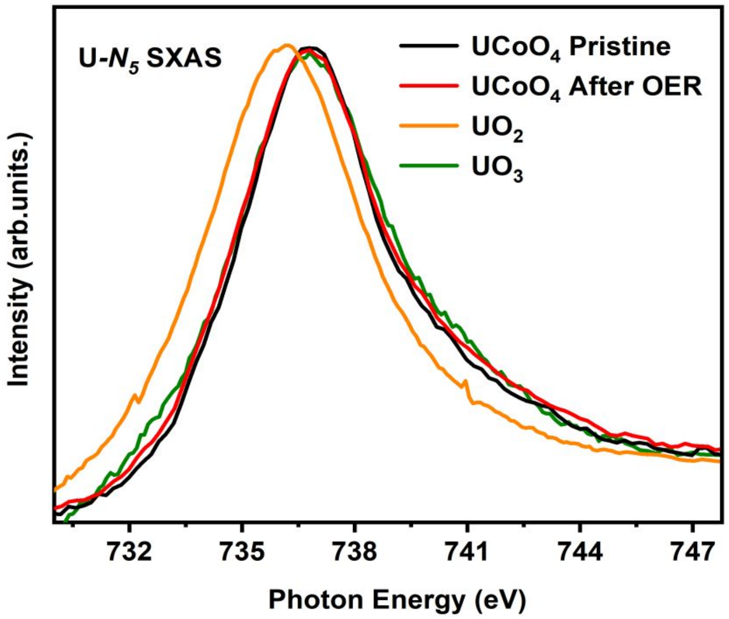

Figure S8. U-N_5 edge SXAS spectra of $UCoO_4$ before and after OER, along with that of UO_2 and UO_3 references.

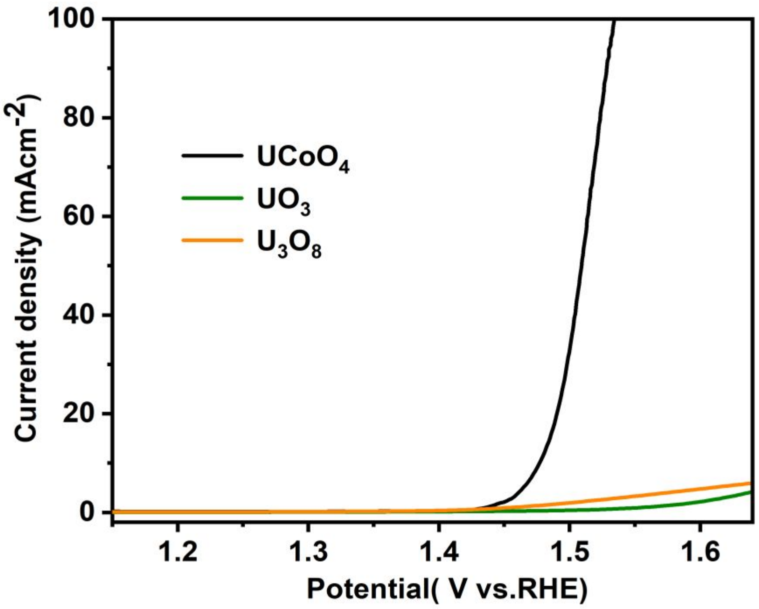

Figure S9. The OER activity of $UCoO_4$, and reference sample U_3O_8, UO_3.

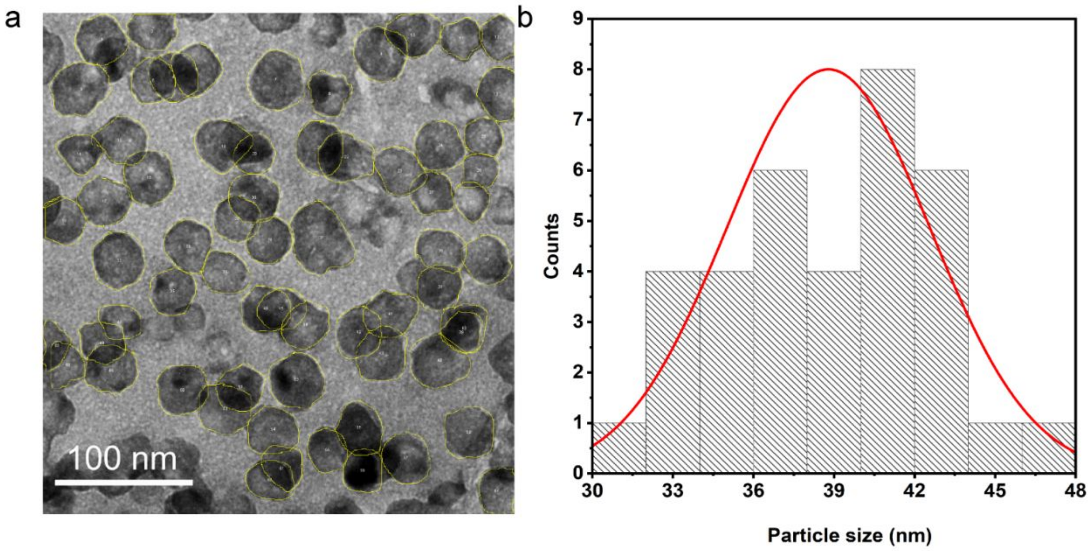

Figure S10. (a) Low magnification TEM image of $UCoO_4$, and **(b)** Distribution diagram for the particle size of $UCoO_4$ powder. The average size of $UCoO_4$ is calculated to be ~39 nm.

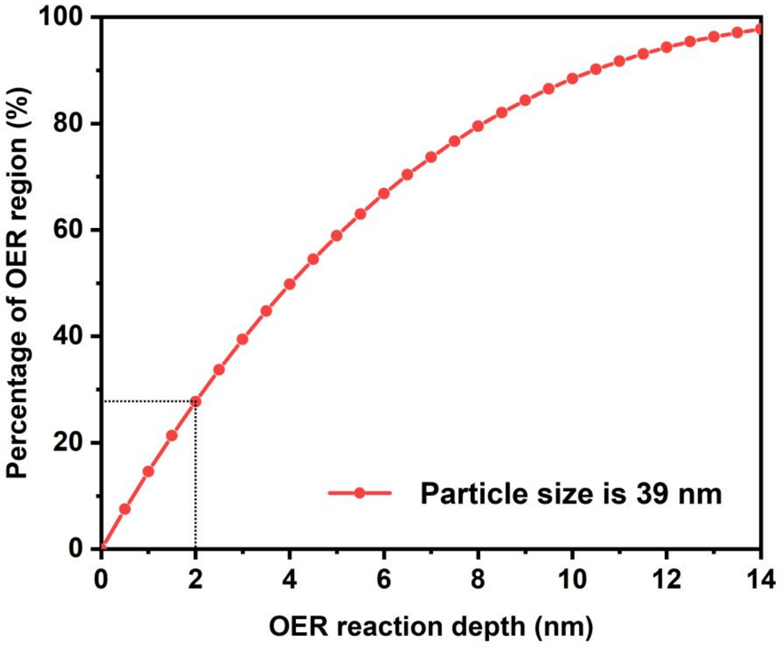

Figure S11. The percentage of OER region signal collected by hard X-ray technology, as a function of OER reaction depth, when the particle size of catalyst is ~39 nm.

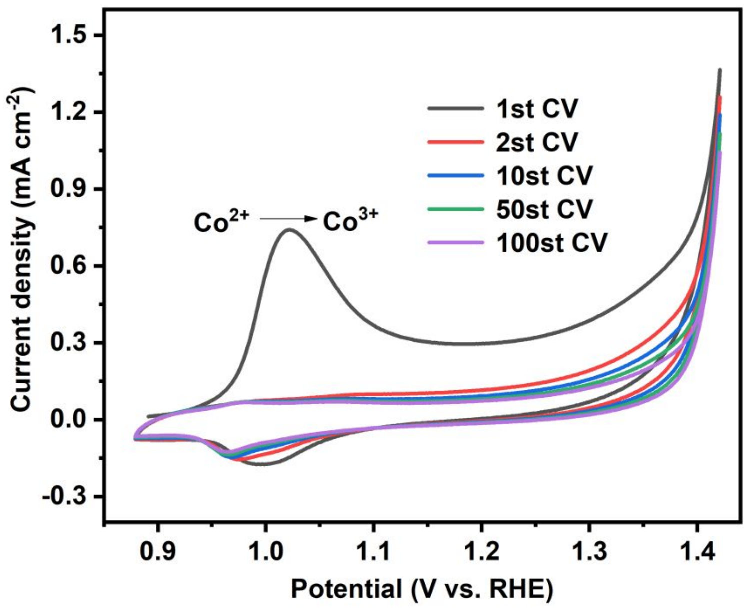

Figure S12. Cyclic voltammograms of the $UCoO_4$ catalyst in 1.0 M KOH electrolyte between 0.88 V and 1.42 V without iR compensation, the scan rate is 10 m Vs^{-1}.

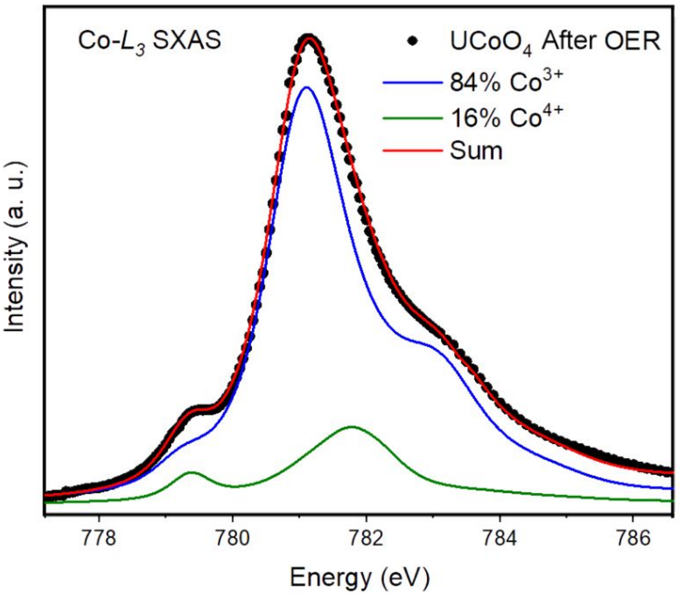

Figure S13. Experimental (dotted black line) and theoretical (red line) spectra of Co-L_3 SXAS spectra of $UCoO_4$ after OER. Theoretical spectra (red line) constructed from a weighted sum of the theoretical simulation for an *LS*-Co^{3+} spectra (blue lines) and an *LS*-Co^{4+} spectra (cyan lines) taken from ref [*Phys. Rev. B,* 2010*,* 81, 115138].

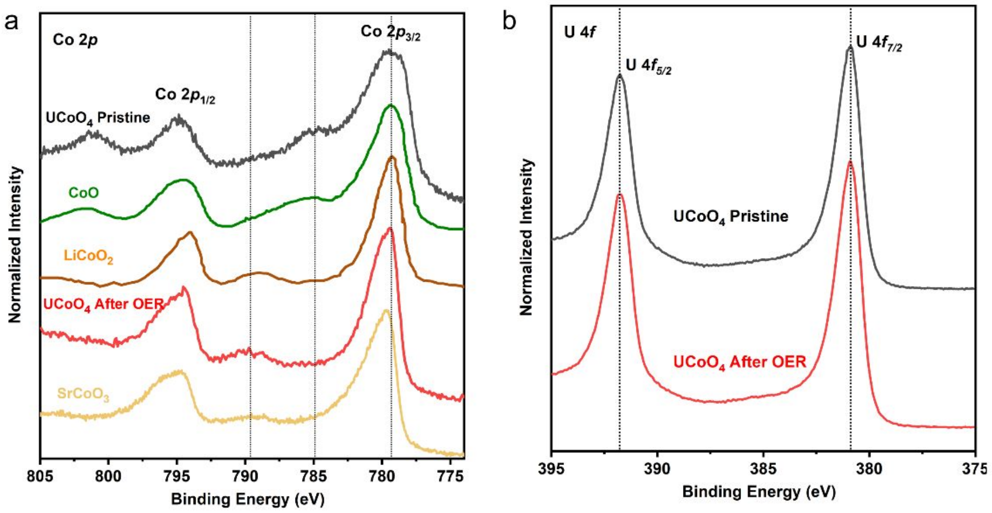

Figure S14. Co-*2p* and U-*4f* spectrum of $UCoO_4$ pristine and after OER. Co 2p of reference CoO and $LiCoO_2$ taken from ref [*Eur. J. Inorg. Chem*. 2017, 587].

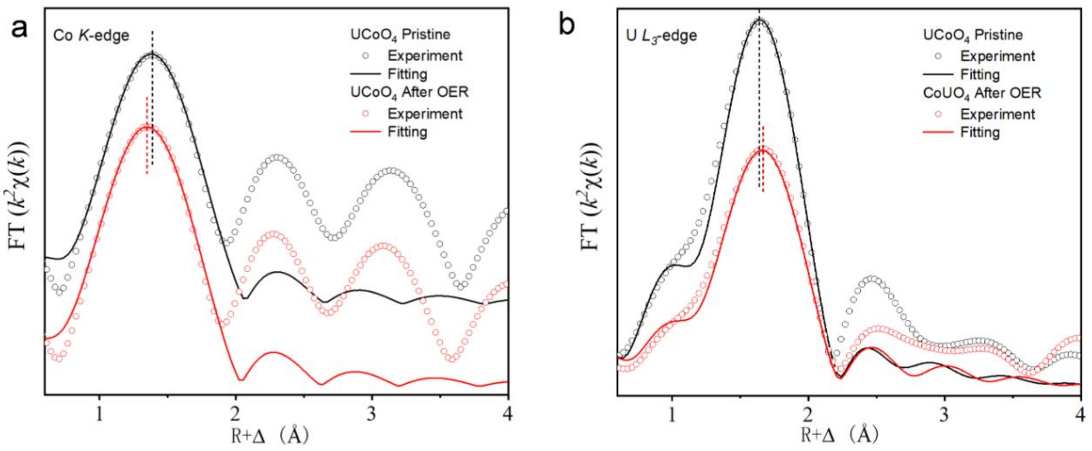

Figure S15. Experimental Fourier transform of the **(a)** Co *K*-edge and **(b)** U L_3-edge EXAFS data of the $UCoO_4$ pristine and after OER, as well as the corresponding fits.

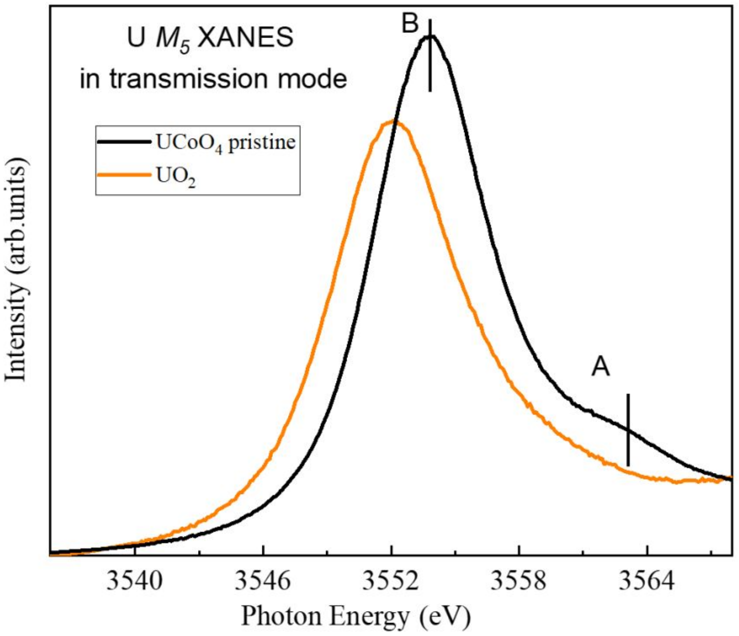

Figure S16. U-M_5 XANES spectra of $UCoO_4$ (black) and UO_2 (orange) collected in the transmission mode using in-house laboratory-based X-ray absorption spectrometer.

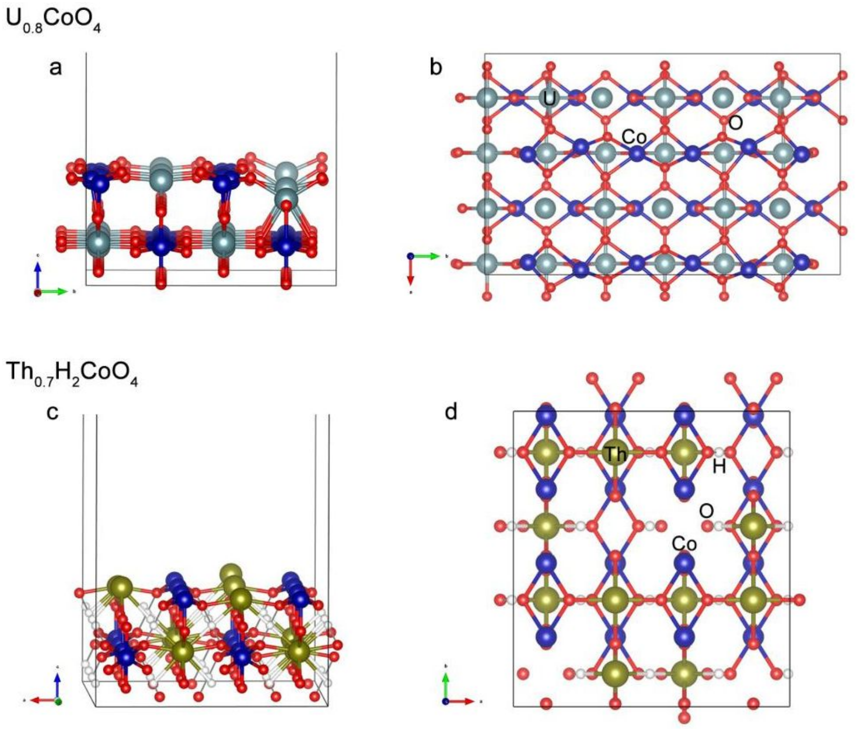

Figure S17. The optimized local structures for the (001) surface. **(a, b)** $U_{0.8}CoO_4$. **(c, d)** $Th_{0.7}H_2CoO_4$. The blue, grey, red, dull yellow, and white balls indicate Co, U, O, Th, and H respectively**.**

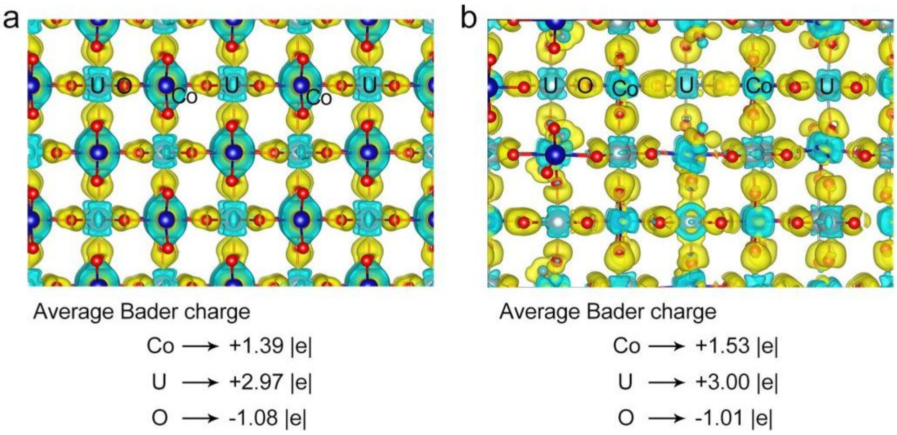

Figure S18. Charge-density differences and Bader charge of **(a)** $UCoO_4$ and **(b)** $U_{0.8}CoO_4$. Blue and yellow regions represent electron depletion and accumulation respectively.

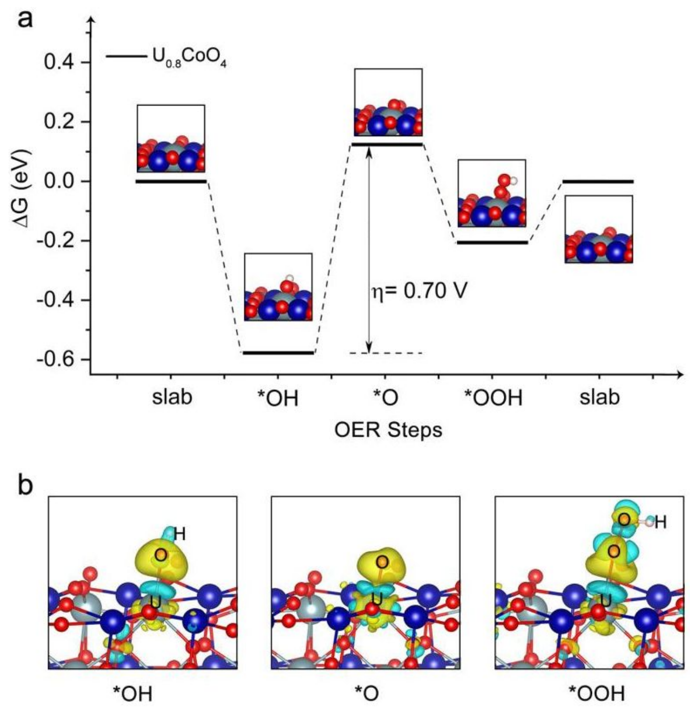

Figure S19. Schematic MAE mechanisms involving four concerted proton-electron transfer steps. **(a)** The free energies at U_{RHE}=1.23 V of OER steps for U atoms in $U_{0.8}Co^{3.2+}O_4$ structural models. **(b)** Charge-density differences of the adsorbed species in MAE scenario. Blue and yellow regions represent electron depletion and accumulation respectively.

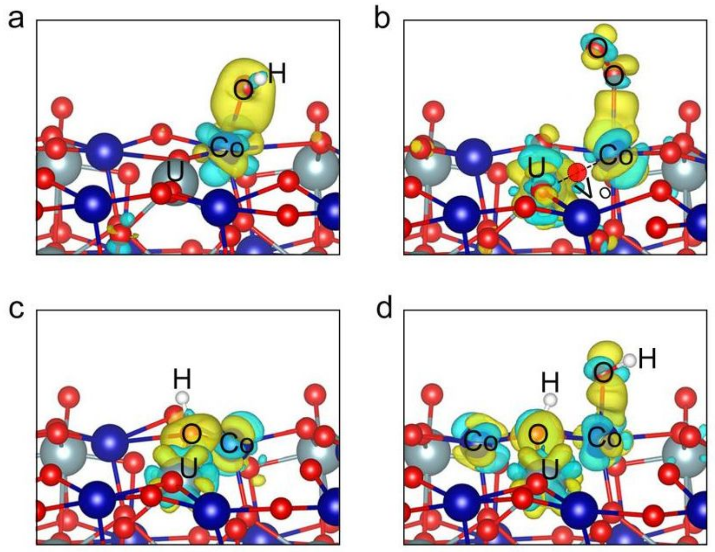

Figure S20. Charge-density differences of four adsorbed species in MLOV scenario. Blue and yellow regions represent electron depletion and accumulation respectively.

Table S1. Evolution of the U/Co ratio in $UCoO_4$ sample as-prepared and after OER reaction at 10 mA cm^{-2} current densities determined by the ICP-MS and SEM-EDS methods.

Methods	Element configuration of U/Co (atom ratio)

	Pristine	Over 1h	Over 6h	Over 12h	Over 24h	Over 48h
SEM-EDS	0.974	0.881	0.861	0.845	0.843	0.843
ICP-MS	0.994	0.912	0.896	0.886	0.862	0.851

Table S2. Parameters of the Fit of the $UCoO_4$ pristine and after OER

**Bond type**	**Sample**	**CN***	**R (Å)**	**σ^2 (10^{-3} Å^2)****	**R factor**
Co-O	Pristine	5.6±0.3	1.96±0.01	15±2	0.017
	After OER	5.5±0.3	1.94±0.01	14±2	0.023
U-O	Pristine	5.1±0.5	2.17±0.01	21±2	0.024
	After OER	4.7±0.4	2.20±0.01	26±2	0.019

*CN: coordination number; S_0^2 was fixed to be 0.70 for Co, and 0.85 for U

**σ^2: Debye−Waller factors

Table S3. The calculated zero-point energy and entropy correction values (in eV) of absorbates in the MLOV mechanism.

	**ZPE**	**TS**	**ZPE-TS**
H_2	0.28	0.40	-0.12
H_2O	0.57	0.58	-0.01
-OH	0.37	0.05	0.32
-OO with V_O	0.18	0.12	0.06
$-OH^{VO}$	0.38	0.05	0.33
$-OH^*$with OH^{VO}	0.70	0.07	0.63

Table S4. Comparison of the electrocatalysic activity of Co-based catalysts toward OER.

**Catalysts**	**Electrolyte**	**Tafel**	**(mV vs. RHE) @ 10mAcm^{-2}**	**Referrences**
$UCoO_4$	1M KOH	47	250	This work
CoO	1M KOH	——	389	*Nano energy* **2019**, 57, 753
Co_3O_4	1M KOH	——	465	*Nano energy* **2019**, 57, 753
$AgCoO_2$	1M KOH	——	395	*Nano energy* **2019**, 57, 753
$Co_{3-x}O_4$	1M KOH	38.2	268	*ACS Catal.* **2018**, 8, 3803
CoOOH	1M KOH	87	253	*J. Mater. Chem. A* **2019**, 7, 23191
$Co(OH)_2/CoOOH$	1M KOH	68	395	*J. Power Sources* **2018**, 396, 395
$LaCoO_3$	1M KOH	180	358	*Chem. Mater.* **2017**, 29, 10534
$Ca_3Co_4O_9$	1M KOH	59	368	*Adv. Mater. Interfaces* **2018**, 5, 1801281

$Zn_{0.35}Co_{0.65}O$	1M KOH	43	322	*Adv. Energy Mater*. **2019**, 9, 1900328
$CuCo_2O_4$	1M KOH	68	290	*ChemElectroChem* **2021**, 8, 135–141
$LiCoO_2$	0.1M KOH	43	280	*J. Mater. Chem.* A **2020**, 8, 19946
$Li_2Co_2O_4$	1M KOH	46	383	*ACS Catal.* **2019**, 9, 7389−7397
$CoMoO_4$	1M KOH	77.4	364	*Inorg. Chem.* **2020**, 59, 17775−17782
$CoWO_4$	1M KOH	89.3	252	*Appl. Surf. Sci.* **2020**, 514,145919
CoV_2O_4	1M KOH	38	300	*ACS Catal.* **2018**, 8, 1259
$CoSnO_3$	1M KOH	145.7	358	*Int. J. Hydrogen Energy* **2019**, 44, 21623
RuO_2	1M KOH	86	206	*Appl. Catal. B* **2021**, 288, 120002
NiO	1M KOH	76.6	340	*Appl. Catal. B* **2021**, 285, 119809
FeO	0.1M KOH	65	308	*J. Mater. Chem. A* **2020**, 8,15140
